# Potentiometric titration for the high precision determination of active components in six types of chemical disinfectants

**DOI:** 10.1371/journal.pone.0203558

**Published:** 2018-09-07

**Authors:** Jiansheng Liang, Junsheng Zhu, Lin Gong, Xiaoli Liu, Bin Wang

**Affiliations:** Department of Disinfection and Pest control, Wuhan Centers for Disease Prevention and Control, Hubei, China; Northeastern University, UNITED STATES

## Abstract

Chemical disinfectants effectively kill pathogenic microorganisms, eliminating routes of transmission for infectious diseases. Accurate quantification of the active ingredients can help make a more effective use of disinfectants. In this study, the active ingredients in six different types of chemical disinfectants were systematically quantified with great precision and accuracy using potentiometric titration. The coefficient of variations fell in the range of 0.04%-0.46%. The recovery rates of samples were all above 95% and the extended uncertainty was 0.32g/L. This method can be broadly applied to the analysis of disinfectants in the future.

## Introduction

Chemical disinfectants effectively kill or remove pathogenic microorganisms, blocking disease transmission routes[1]. Commonly used chemical disinfectants include chlorine compounds, iodine, oxidants, aldehydes, alcohols, and quaternary ammonium salts[2]. The efficacy of these chemical disinfectants to eliminate microbes can be affected by many factors[3], of which the content of the active component of the disinfectant is clearly the most critical one. The most commonly used techniques for determining the content of chemicals include volumetric analysis, spectrophotometry, gas chromatography, and high-performance liquid chromatography[4–7]. Nevertheless, using any of the above methods for systematic measurement of the components in a chemical disinfectant can be problematic due to certain limitations of the techniques, such as large error range, difficulty of operation, susceptibility to interference, and the different properties of the various chemical components contained within each disinfectant. In this article, potentiometric titration[8]is used to determine the effective content of general chemical disinfectants from six different categories. The method offers many advantages, including simplicity, speed, easier end-point determination and higher accuracy. The testing and results of potentiometric titration are discussed in the following sections.

## Materials and methods

### Instruments and reagents

#### A. Instruments

Titrations were performed using an 809 Titrando automatic potentiometric titrator, a burette drive and a magnetic stirrer.

#### B. Test samples

The test samples included chlorine compounds (Dezhou Gelijie Disinfection Products Limited Company, lot number:20150722,available chlorine:4.5%-5.5%), iodine (Wuhan Lianhua Sanitary Supplies Factory, lot number:20161004,available iodine:1.0%-1.2%), hydrogen peroxide (Wuhan Lianhua Sanitary Supplies Factory, lot number:20160902, available hydrogen peroxide: 2.8%-3.5%), glutaraldehyde (Wuhan Donghuxing Technology Limited Company, lot number:20151103,available glutaraldehyde:2.0%-2.2%), chlorhexidine (Dezhou Gelijie Disinfection Products Limited Company, lot number:20161115,available chlorhexidine acetate:94%-96%) and benzalkonium bromide (Wuhan Donghuxing Technology Limited Company, lot number:20160727,available benzalkonium bromide:0.9%-1.1%). These samples were all used prior to the expiration date.

#### C. Reagents

The following aqueous solutions were prepared: sulfuric acid (lot number:T20140412, 2mol/L,),potassium iodide (lot number:F20150311, 100g/L), manganese sulfate (lot number:F20150616, 100g/L), triethanolamine (lot number:F20130616, 65g/L), hydrochloric acid (lot number:20140208, 10g/L),sodium hydroxide (lot number:20120919,43g/L) and acetic acid (lot number:T20130207, 36%).Glacial acetic acid (lot number:20130202,≥99.5%) and acetone (lot number:T20141103, ≥99.5%)were also used.They were all purchased from Sinopharm Chemical Reagant Limited Company, A neutral hydroxylamine hydrochloride solution was prepared by adding 17.5g of hydroxylamine hydrochloride to 75mL of distilled water and diluting to 500mL with isopropyl alcohol. Next,15 mL of a bromophenol blue/ethanol solution(0.4g/L) was added, and finally triethanolamine(65g/L) was added to the solution until a blue-green color was obtained.

### Electrode measurements

Composite platinum (Pt) electrodes or a monomer platinum (Pt) indicator electrode and reference electrode were used to measure available chlorine, available iodine and hydrogen peroxide content.A composite water phase pH electrode(containing a pH indicator electrode and a Ag/AgCl reference electrode)or an aqueous phase pH-indicating electrode and reference electrode monomer were used to measure glutaraldehyde content.A composite non-aqueous phase pH electrode (containing a pH indicator electrode and a Ag/AgCl reference electrode)was used to measure the chlorhexidine content. Finally, a surfactant aqueous phase titration indicator electrode and a reference electrode were used to measure benzalkonium bromide content.

### Test method

#### A. Ambient temperature and humidity

The ambient temperature was 20°C-25°C,and the relative humidity was 45%-85% for these studies.

#### B. Principle

The indicator electrode and the reference electrode (or the reference and an indicator electrode included as a composite electrode) were immersed in the same solution, in which the reference electrode was maintained at a constant; then,the indicator electrode was immersed in the test substance. When the titration approached the equivalence point, small changes in the activity of the test substance solution elicited a dramatic change to the indicator electrode, and the largest change detected in indicator electrode potential was considered the end point of titration.

#### C. Titration mode, control program design, and installation

The Tiamo operating procedure software was installed before installing and debugging the automatic potentiometric titrator.Prior to using the instrument,titration mode was selected,and the standard detection method for test items and parameters were entered into the control program. The test project and the indicators for samples were transferred to the method bar and bar usage before conducting the titration,allowing the database to generate results automatically.

### Method for determination

#### A.Capacity Analysis

This method was performed in accordance with the Ministry of Health of the People's Republic of China’s“Disinfection Technical Specifications” (2002) [9].

#### B. Automatic potentiometric titration (dx.doi.org/10.17504/protocols.io.rkqd4vw)

This method was performed as described in GB/T 9725–2007 “Chemical Reagent Potentiometric Titration General Principles” (ISO6353-1:1982)[10] and in accordance with DB/T 801–2012,“Determination of the Active Ingredient in the Chemical Disinfectant Automatic Potentiometric Titration”[11].

Method details: (1) The electrode and the titration mode were chosen according to the type of sample.(2) Sample pretreatment: 10 times the amount of solid (powder, tablet) chemical disinfectant required for analysis was obtained, and,the appropriate amount of sample for accurate determination after grinding was weighed. The liquid chemical disinfectant was shaken until reaching a uniform state and then analyzed with or without dilution.(3) The sample information, standard titrant concentration and formula were entered as the input into the device,and the content of the effective component of the sample was measured.(4)The database then generated the results automatically.

#### C. Determination of the titration equivalence point

**Fig 1:**
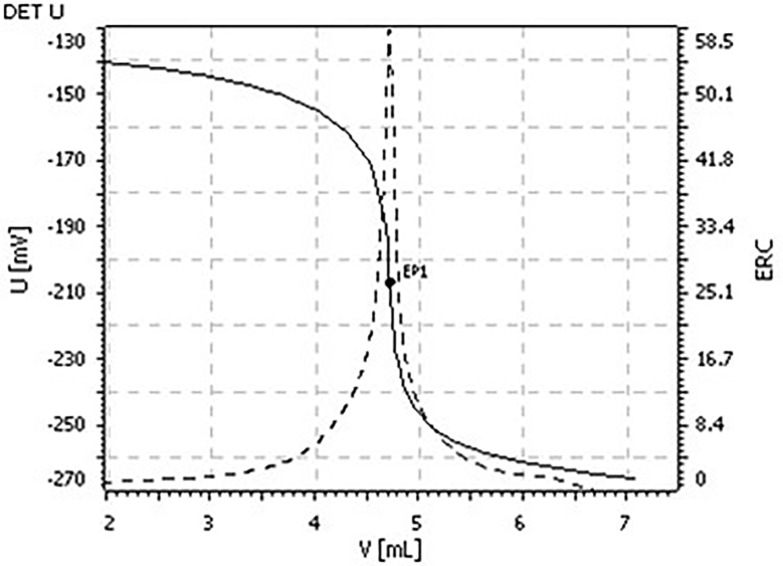
Potentiometric titration curve. The solid curve represents the change in electric potential and its first derivative is plotted as the dashed curve.

A peak maximum appears during the titration procedure when the threshold value is exceeded, and the jump point, or the titration equivalence point, is identified by taking the first derivative of the curve.The titration equivalence point corresponding to the volume of titrant recorded (a titration curve example is shown in Fig 1) is transferred to the formula for further calculations.

#### D. Calculation of results

The content of the test substance was determined using the chemical reaction formula and the amount of titrant consumed according to formulas (1) and (2).The result was averaged across six experiments.

Liquid chemical disinfectant effective content

$$X(g/L)=\frac{c\times Vst\times k}{V}\times 1000$$ (1)

Solid chemical disinfectant effective content

$$X(\%)=\frac{c\times Vst\times k}{m}\times 100\%$$ (2)

For formulas(1) and (2): X = substance content (g/L or %), C = standard titration solution concentration (mol/L),VST = corrected volume for the standard titration (mL), k = coefficient (g), V = test substance sampling volume (mL), and m = test substance sampling mass(g).

### Evaluation and methods

#### A. Precision assessment

The measurements were compared with those obtained by the manual chemical titration method (i.e., the volumetric analysis)The precision of the method was assessed by the coefficient of variation (CV) according to formulas(3) and (4), which should not be greater than 1.0%.

$$S=\sqrt{\frac{\sum {\left(x_{i}‑x_{t}\right)}^{2}}{n‑1}}\;(=1,2\cdots ,n)$$ (3)

$$CV=\frac{S}{x_{t}}\times 100\%$$ (4)

**in which** S = standard deviation, X_i_ = single measured value, X_t_ = average of measurements, Σ = the sum of absolute values, CV = coefficient of variation, and n = number of measurements.

#### B. Linearity test

For the six types of chemical disinfectants that were analyzed,available chlorine(3.35–42.97g/L), available iodine (2.29–11.43g/L),hydrogen peroxide (10.48–34.94g/L),glutaraldehyde (4.57–19.99g/L), benzalkonium bromide(0.83–8.50g/L)and chlorhexidine acetate(7.74%-93.41%)were determined by titration with sodium thiosulfate (0.1mol/L), potassium permanganate(0.02mol/L),sulfuric acid (0.25mol/L), perchloric acid (0.1mol/L) and sodium tetraphenylborate (0.02mol/L), respectively. The measured chemical disinfectant composition was plotted as the horizontal axis, and the consumption of titration liquid volume was plotted as the vertical axis. A linear regression analysis was performed to evaluate the reproducibility of the method. The linear regression equation: y = a+bx (y: titrant consumption, a: constant, b: slope, x: content or sample volume).

#### C. Accuracy assessment

The accuracy of the method was measured by the recovery of standard substance added (P) as calculated by Eq (5), which should be>95%.

$$P=\frac{c2-c1}{c3}\times 100\%$$ (5)

**in which** P = spiked recoveries, c1 = sample background concentration, c2 = sample spiked concentration, and c3 = plus scalar.

#### D. Uncertainty assessment

The main sources of uncertainty of this analysis included sample volume(μ_1_), standard solution preparation(μ_2_), repeated measurements (μ_3_)and interpolation using the standard curve(μ_4_). Glutaraldehyde disinfectant was selected as a substance to estimate the upper bound of the uncertainty of this method because of the complexity of its composition.

Mathematical model

$$X(g/L)=\frac{c\times Vst\times 0.1001}{V}\times 1000$$ (6)

**in which** x = Glutaraldehyde content(g/L), c = concentration of sulfuric acid titrant(mol/L), Vst = corrected volume of sulfuric acid titrant(ml), and 0.1001 is the conversion factor.

### Statistical analysis

The data from the experiments were analyzed with the SAS9.0 statistics software. Results from two groups were compared using independent sample T-test and correlation analysis. The difference was considered to be statistically significant with a P≤0.05.

## Results

### Accuracy and precision of potentiometric titration

The results of potentiometric titration and direct titration were shown in Table 1. there were no significant differences between the two methods (P>0.05).This indicated that the analysis of chemical disinfectants by potentiometric titration agrees with the current National Standard (direct titration). the CVs for the effective content in chemical disinfectants determined by potentiometric titration were lower than those obtained using the other method, except for the bromogeramine disinfectant, suggesting that potentiometric titration has a superior precision direct titration.

**Table 1:** Results and CVs of the effective content of six different chemical disinfectants by potentiometric titration and direct titration.

Experimental projects(unit)	Experimental methods^a^	Each experimental result						Mean	Standard deviation	CV(%)	P value
		1	2	3	4	5	6				
Available chlorine concentration(g/L)	A	43.34	43.44	43.36	43.35	43.38	43.41	43.38	0.035	0.081	0.1013
	B	43.16	43.18	43.13	43.17	43.19	43.16	43.17	0.019	0.044	
available iodine concentration (g/L)	A	11.41	11.43	11.41	11.43	11.43	11.43	11.42	0.010	0.083	0.4956
	B	11.42	11.43	11.43	11.42	11.43	11.43	11.43	0.005	0.043	
hydrogen peroxide concentration (g/L)	A	35.04	34.92	34.95	34.96	34.96	34.96	34.97	0.036	0.104	0.1116
	B	34.90	34.93	34.94	34.94	34.96	34.93	34.94	0.018	0.052	
glutaraldehyde concentration (g/L)	A	19.84	19.71	19.89	19.71	19.84	19.75	19.79	0.070	0.354	0.1274
	B	20.02	20.04	20.01	20.04	20.03	20.01	20.03	0.013	0.064	
Chlorhexidine acetate concentration (%)	A	94.47	95.05	94.62	95.03	94.67	94.57	94.74	0.224	0.236	0.6414
	B	94.87	94.96	94.84	94.56	94.87	94.65	94.79	0.139	0.147	
Benzalkonium bromide concentration (g/L)	A	8.43	8.40	8.49	8.43	8.46	8.49	8.45	0.033	0.392	0.0795
	B	8.48	8.54	8.51	8.54	8.43	8.47	8.50	0.040	0.465	

^a^ A = direct titration, B = potentiometric titration.

### Linearity potentiometric titration tests

**Fig 2:**
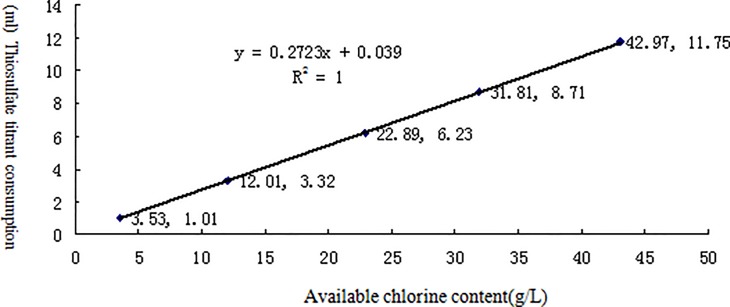
Linear regression diagram for available chlorine and its neutralizer.

**Fig 3:**
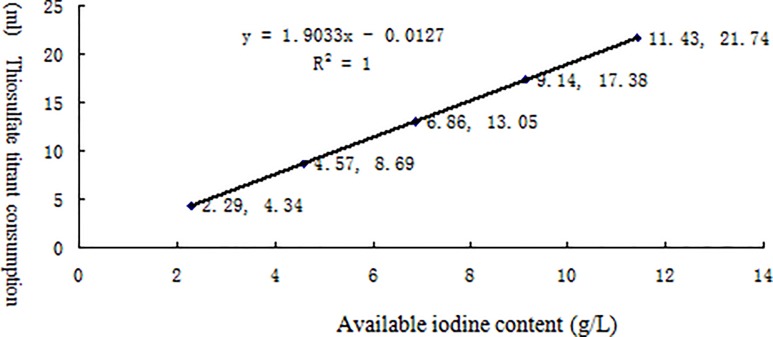
Linear regression diagram for available iodine and its neutralizer.

**Fig 4:**
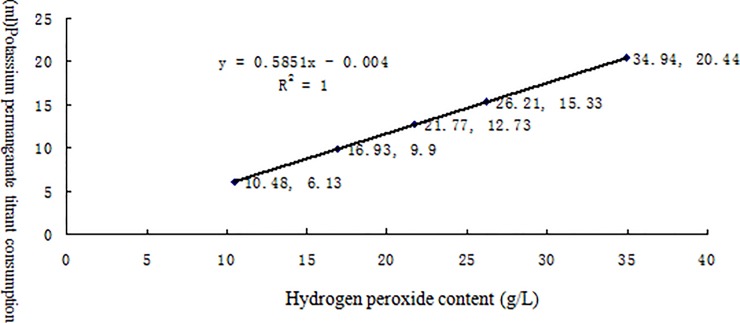
Linear regression diagram for hydrogen peroxide and its neutralizer.

**Fig 5:**
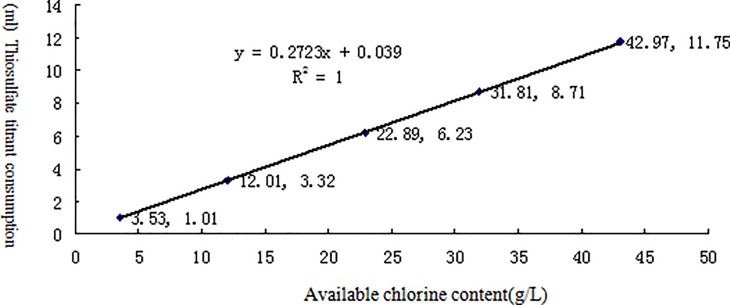
Linear regression diagram for glutaraldehyde and its neutralizer.

**Fig 6:**
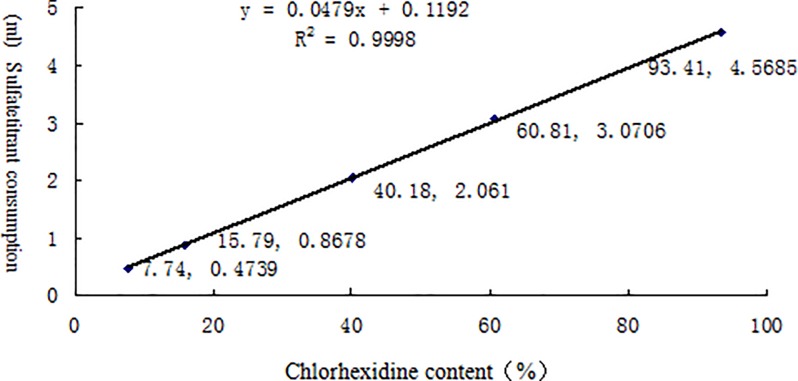
Linear regression diagram for chlorhexidine and its neutralizer.

**Fig 7:**
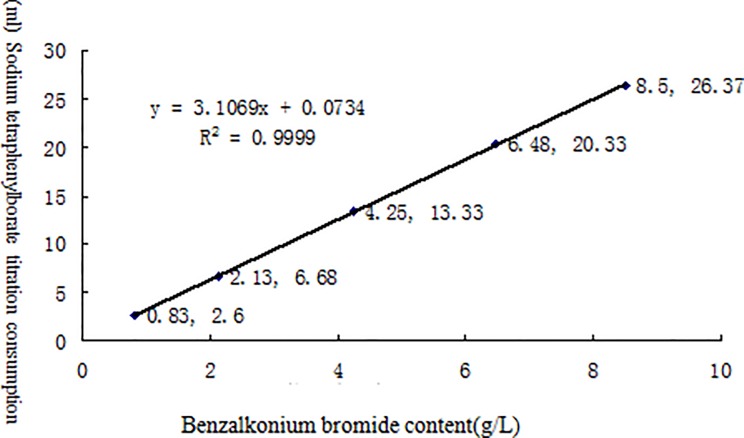
Linear regression diagramfor benzalkonium bromide and its neutralizer.

Table 2 lists the linearity test results for the effective contents of six different chemical disinfectants measured by potentiometric titration. The results for each disinfectant and its neutralizer are shown in Figs 2–7 along with the linear regression models. These equations were as follows: for available chlorine, **y = 0.2723x + 0.039** and the R^2^ (determinate coefficient) = 1. 000; for available iodine, **y = 1.9033x − 0.0127**, R^2^ = 1.000;for hydrogen peroxide, **y = 0.5851x − 0.004**, R^2^ = 1.000;for glutaraldehyde, **y = 0.3993x + 0.0046**, R^2^ = 1.000; for chlorhexidine acetate, **y = 0.0479x + 0.1192**, R^2^ = 0.9998, and for benzalkoniumbromide, **y = 3.1069x + 0.0734**, R^2^ = 0.9999. The above coefficients were all greater than 0.999, suggesting a strong linearity of the potentiometric titration method in analyzing the active ingredients of all these disinfectants.

**Table 2:** Linear test data for the effective contents of six different chemical disinfectants measured by potentiometric titration(n = 5).

Experimental project (Content/sample volume)	Reagents andneutralizer^a^	groups				
		1	2	3	4	5
Available chlorine	Available chlorine (g/L)	3.53	12.01	22.89	31.81	42.97
	Thiosulfate titrant	1.01	3.32	6.23	8.71	11.75
Available iodine	Available iodine (g/L)	2.29	4.57	6.86	9.14	11.43
	Thiosulfate titrant	4.34	8.69	13.05	17.38	21.74
Peroxide	Hydrogen peroxide (g/L)	10.48	16.93	21.77	26.21	34.94
	Potassium permanganate titrant	6.13	9.90	12.73	15.33	20.44
Glutaraldehyde	Glutaraldehyde (g/L)	4.57	9.63	14.64	17.38	19.99
	Sulfate titrant	1.83	3.85	5.85	6.94	7.99
Chlorhexidine	Chlorhexidine content (%)	7.74	15.79	40.18	60.81	93.41
	Perchloric acid titrant	0.47	0.87	2.06	3.07	4.57
Benzalkonium bromide	Benzalkonium bromide(g/L)	0.83	2.13	4.25	6.48	8.50
	Sodium tetraphenylborate titration	2.60	6.68	13.33	20.33	26.67

^a^ All of the reagent volumes were 1mL,all neutralizer units are mL.

### Recovery tests of standard substance (sample) addition

The accuracy of potentiometric titration was assessed by the recovery rates of standard substance (sample).If the recovery was between 90% and 110%,the method was considered reliable. The data in Table 3 show that the recovery rates for all of the disinfectants were in the range of 95%-104%, demonstrating the high reliability of this method.

**Table 3:** The recovery test results of standard substance (sample) addition in six types of chemical disinfectants.

Disinfectants	Grouping (g/L)	Measurement results (Active ingredient content of each the preparation solution(g/L))						Mean (g/L)	Recoveries(%)
		1	2	3	4	5	6		
Chlorine	Background	9.34	9.33	9.34	9.36	9.31	9.34	9.34	/
	4.30	13.62	13.59	13.63	13.62	13.61	13.58	13.61	99.30
	8.60	17.91	17.94	17.89	17.92	17.91	17.91	17.91	99.65
	12.89	22.25	22.20	22.20	22.22	22.20	22.19	22.21	99.84
Iodine	Background	1.16	1.15	1.16	1.17	1.14	1.17	1.16	/
	2.29	3.53	3.52	3.51	3.52	3.50	3.51	3.52	103.0
	4.58	5.90	5.93	5.89	5.91	5.89	5.88	5.90	103.5
	9.16	10.35	10.34	10.32	10.33	10.34	10.31	10.33	100.1
Hydrogen peroxide	Background	11.45	11.44	11.44	11.46	11.44	11.44	11.45	/
	3.49	14.95	14.96	14.96	14.95	14.96	14.96	14.96	100.6
	6.99	18.42	18.41	18.41	18.42	18.43	18.41	18.42	99.71
	10.48	21.90	21.91	21.94	21.94	21.89	21.90	21.91	99.81
Glutaraldehyde	Background	9.88	9.85	9.81	9.79	9.83	9.87	9.84	/
	2.02	11.87	11.81	11.79	11.85	11.81	11.83	11.83	98.51
	4.03	13.85	13.96	13.91	13.87	13.95	13.89	13.91	101.0%
	10.08	19.97	19.85	19.85	19.95	19.92	19.88	19.90	99.80%
Chlorhexidine	Background	0.0968	0.0960	0.0972	0.0960	0.0972	0.0960	0.0965	/
	0.0186	0.1145	0.1144	0.1147	0.1146	0.1145	0.1147	0.1146	97.31
	0.0284	0.1234	0.1237	0.1234	0.1238	0.1234	0.1231	0.1235	95.07
	0.0379	0.1336	0.1339	0.1341	0.1336	0.1339	0.1342	0.1339	98.68
Benzalkonium bromide	Background	0.85	0.84	0.85	0.84	0.84	0.84	0.84	/
	1.7	2.51	2.49	2.52	2.48	2.48	2.49	2.49	97.06
	3.4	4.22	4.26	4.23	4.22	4.21	4.23	4.23	99.71
	6.8	7.66	7.68	7.62	7.65	7.68	7.63	7.65	100.1

### Uncertainty measurements for glutaraldehyde disinfectant

#### A. Relative uncertainty caused by sampling volume (μ_1_)

By sampling 10L with a 10mL single scale line straw, the relative uncertainty (μ_1_) caused by the sampling volume (v_1_)was the result of a reading value variation from the scale line(μ_1.1_) and the volume variation of the scale due to a temperature change(μ_1.2_).

1. The allowable error in a 10mL single scale line straw was 0.1mL, with a uniform distribution of $\kappa =\sqrt{3}$, then:
  $$\mu_{1.1}=\frac{\frac{U_{1.1}}{\kappa }}{V_{1}}=\frac{\frac{0.1}{\sqrt{3}}}{10}=0.577\%$$
2. Set temperature variation was 3°C (ΔT = 3,expansion coefficient of water **α** = 2×10^−4^/°C, with a uniform distribution of $\kappa =\sqrt{3}$, then:
  $$\mu_{1.2}=\frac{\Delta T\times \alpha }{\kappa }=\frac{3\times 2\times 10^{-4}}{\sqrt{3}}=0.0346\%$$
3. Relative uncertainty (μ_1_) caused by sampling volume (v_1_)
  $$\mu_{1}=\sqrt{{{\mu_{1.1}}^{2}+\mu_{1.2}}^{2}}=\sqrt{{0.577\%}^{2}+{0.0346\%}^{2}}=0.578\%$$

#### B.Relative uncertainty caused by preparation of the standard titration solution (μ_2_)

1 The allowable error in a 10mL single scale line straw was 0.1mL,with a uniform distribution of $\kappa =\sqrt{3}$, then:
  $$\mu_{2.1}=\frac{\frac{U_{2.1}}{\kappa }}{V_{1}}=\frac{\frac{0.1}{\sqrt{3}}}{10}=0.577\%$$
2 Uncertainty due to the burette (μ_2.2_)
  The value of a scale division in a 50mL acid burette was 0.1 mL,and the maximum permissible error of which was ±0.05mL,with a uniform distribution of $\kappa =\sqrt{3}$ and a half width **α** = 0.05 mL, then:
  $$\mu_{2.2}=\frac{\frac{\alpha }{\kappa }}{V_{2}}=\frac{\frac{0.05}{\sqrt{3}}}{50}=0.0577\%$$
3 The concentration uncertainty of calibrated standard titrant solution by means of repetitive measurements(μ_2.3_).

The same standard solution was titrated 6 times,and the results are shown in Table 4.

**Table 4:** Calibration results for titration of standard sulfuric acid solution.

Calibration number	x_i_ (mol/L)	$\bar{x}$ (mol/L)	S(x) (%)	μ_2.3_ (%)	n-1
1	0.2521	0.2530	0.00392	0.0016	5
2	0.2537				
3	0.2536				
4	0.2525				
5	0.2529				
6	0.2532				

$$\bar{x}=\frac{1}{n}\sum_{i=1}^{6}x_{i}=0.2530$$

$$S(x)=\sqrt{\frac{\sum_{i}^{6}{\left(x_{i}-\bar{x}\right)}^{2}}{n-1}}=0.0000392=0.00392\%$$

$$\mu_{2.3}=\frac{S(x)}{\sqrt{n}}=\frac{0.0000392}{2.4494897}=0.0016\%$$

4 Relative uncertainty caused by the preparation of standard titration solutions (μ2)
  $$\mu_{2}=\sqrt{{\mu_{2.1}}^{2}+{\mu_{2.2}}^{2}+{\mu_{2.3}}^{2}}=\sqrt{{0.577\%}^{2}+{0.0577\%}^{2}+{0.0016\%}^{2}}=0.580\%$$

#### C. Relative uncertainty caused by the repeatability of the results (μ_3_)

Samples were measured 6 times in parallel,and the results for glutaraldehyde content are listed in Table 1:

$$\mu_{3}=\frac{S}{\sqrt{n}\times \bar{x}}=0.0265\%$$

#### D. Relative uncertainty caused by the standard curve(μ_4_)

From Fig 5, the glutaraldehyde content curve was analyzed by linear regression, andthe linear regression equation was:y = 0.3993x+0.0046, R = 1.000.

1. The standard deviation of the regression line (μ_4.1_)
  $$\mu_{4.1}=\sqrt{\frac{\sum_{j=1}^{m}\sum_{i=1}^{n}{\left[Y_{ij}-(a+bc_{i})\right]}^{2}}{m\times n-2}}=\sqrt{\frac{3.16754\times 10^{-5}}{1\times 5-2}}=3.249\times 10^{-3}$$
  in which n = the concentration point(n = 5), m = the repeat measurement frequency for each concentration point (m = 1)
2. The glutaraldehyde concentration sum of squares (Ss)
  $$SS=\sum {\left(c_{i}-\bar{c}\right)}^{2}=145.591$$
3. Relative uncertainty caused by the standard curve(μ_4_)
  $$\begin{array}{l} \mu_{4}=\frac{\mu_{4.1}}{C_{X}\times b}\sqrt{\frac{1}{m}+\frac{1}{n}+\frac{{\left(c_{x}-\bar{c}\right)}^{2}}{ss}}\\ \;=\frac{3.249\times 10^{-3}}{20.03\times 0.3993}\times \sqrt{\frac{1}{1}+\frac{1}{5}+\frac{{\left(20.03-13.242\right)}^{2}}{145.591}}\\ \;=0.05\% \end{array}$$
  in which concentration of sample C_X_ = 20.03g/L

#### E. Synthetic relative uncertainty(μ_w_)

Each relative uncertainty was independent; therefore, the synthetic relative uncertainty could be calculated as follows:

$$\begin{array}{l} \mu_{w}=\sqrt{{\mu_{1}}^{2}{{+\mu }_{2}}^{2}{{+\mu }_{3}}^{2}{{+\mu }_{4}}^{2}}\\ \;=\sqrt{{0.578\%}^{2}{{+0.580\%}_{1}}^{2}+{0.0265\%}^{2}+{0.05\%}^{2}}\\ \;=0.8208\% \end{array}$$

The synthetic standard uncertainty μ(w):

$${\mu (w)=\bar{x}\times \mu }_{w}=20.03\times 0.008208=0.16g/L$$

#### F. Expanded uncertainty (U) and the result of sample concentration (C)

Setting the coverage factor k = 2, the extended uncertainty:

U=k×μ(w)=2×0.16g/L=0.32g/L

The result is expressed as follows:

$$CC=\bar{x}\bar{x}\pm UU=(20.03\pm 0.32)g/L$$

**G.** By analyzing and calculating the uncertainties for the glutaraldehyde disinfectant,all of the partial uncertainties were very low(<0.60%),and the value for the expanded uncertainty was 0.32g/L.This suggests that potentiometric titration measurements are of high quality.

## Discussion

In classical chemical titration (volumetric analysis), the endpoint is based on the color change of an indicator. If the sample being tested is colored or turbid, it is difficult to estimate the titration endpoint with a reliable indicator. In contrast, potentiometric titration relies on abrupt changes in electrode potential to determine the endpoint. The ion concentration being evaluated often varies by orders of magnitude and generates abrupt changes in the electrode potential when the endpoint is approached. The content of the samples can then be calculated from titrant consumption.

In this article, potentiometric titration was adopted to quantify the effective contents of 6 different chemical disinfectants. Compared to standard chemical titration,this method offers many advantages, including simplicity, speed, ease of end-point determination, accuracy, stability, and the ability to overcome a number of confounding factors. Therefore,potentiometric titration is suitable for the fast and accurate determination of 1) effective chlorine/effective iodine/hydrogen peroxide content using the selective composite platinum (Pt) electrode or monomer platinum (Pt) indicator electrode combined with reference electrode, 2) glutaraldehyde content using the composite aqueous phase acid-base electrode (containing pH indicator electrode and Ag/AgCl Reference electrode) or aqueous phase acid-base monomer indicator electrode combined with reference electrode, 3) chlorhexidine acetate using the composite non-aqueous phase acid-base electrode (containing pH indicator electrode and Ag/AgCl reference electrode), and 4) benzalkonium bromide using the surfactant aqueous phase titration indicator electrode and the reference electrode. This method is particularly adaptable to determining the content of certain bactericidal active ingredients in compound chemical disinfectants.

This method has robust precision for all the disinfectants tested, with the CVs ranging from 0.04% to 0.46% Furthermore, our tests also show that the potentiometric titration method yields results that agree well with the current National Standard (direct titration) while having outstanding accuracy as indicated by high the recovery rates (greater than 95% for all samples tested).

The results of the linearity tests shows that potentiometric titration maintains an excellent linearity for a wide concentration range of the analyte. Moreover, the results also demonstrate good robustness.The value for expanded uncertainty was only 0.32g/L. This work represents the first analysis of various chemical disinfectants for systematic determination of the effective contents using potentiometric titration. The robustness and simplicity of this method will prepare it for a broad application in the analysis of disinfectants in the future.
